# Supplementary material for: Emiliania huxleyi—Bacteria Interactions under Increasing CO2 Concentrations
Source: Microorganisms. 2022 Dec 13;10(12):2461. doi: 10.3390/microorganisms10122461 (PMC9786219; doi:10.3390/microorganisms10122461)
Supplement: Supplementary file 1 [file microorganisms-10-02461-s001.zip › microorganisms-2043679-supplementary/microorganisms-2043679-supplementary.pdf]

Supplementary Tables and Figures

Table S1. Carbonate chemistry at the beginning, after 4 days of incubation and through (average) the experiments.

Sample	pHt	TA (μmol kg^{-1})	*p* CO_2 (μatm)	Avg *p* CO_2 (μatm)	Avg *p* CO_2 (μatm)	DIC (μmol kg^{-1})
Media Present	7.99	2388	475			2137
Media Future	7.69	2377	1056			2265
Present						
Ehux 1	7.96	2347	513	494		2117
Ehux 2	7.97	2321	493	484		2087
Ehux 3	7.97	2321	494	485	487	2088
B 1	7.94	2383	549	512		2160
B 2	7.95	2371	532	504		2144
B 3	7.93	2369	552	513	510	2149
I 1	7.93	2377	562	519		2160
I 2	7.93	2375	560	517		2158
I 3	7.94	2372	537	506	514	2147
Ehux + B 1	7.94	2350	534	505		2127
E hux+ B 2	7.96	2342	511	493		2112
Ehux + B 3	7.97	2348	492	483	494	2110
Ehux + I 1	7.96	2362	518	497		2132
Ehux + I 2	7.96	2335	503	489		2103
Ehux + I 3	7.97	2346	499	487	491	2111
Future						
Ehux 1	7.89	2269	597	827		2077
Ehux 2	7.88	2263	610	833		2076
Ehux 3	7.87	2285	631	844	835	2101
B 1	7.75	2377	907	982		2242
B 2	7.74	2374	914	985		2239
B 3	7.76	2387	876	966	978	2245
I 2	7.76	2382	882	969		2242
I 3	7.76	2378	886	971	970	2239
Ehux + B 1	7.82	2304	721	889		2140
E hux+ B 2	7.90	2265	583	819		2070
Ehux + B 3	7.83	2293	695	875	861	2123
Ehux + I 1	7.81	2308	742	899		2148
Ehux + I 2	7.87	2261	627	842		2079
Ehux + I 3	7.85	2279	657	857	866	2102

Table S2. pH_t after 13 / 14 days of incubation.

end bloom phase

	Present	Future
Ehux 1	8.20	8.02
Ehux 2	8.21	8.03
Ehux 3	8.12	8.02
B 1	7.94	7.79
B 2	7.94	7.76
B 3	7.93	7.78
I 1	7.91	
I 2	7.98	7.76
I 3	7.94	7.78
Ehux + B 1	8.27	8.07
E hux+ B 2	8.27	8.08
Ehux + B 3	8.29	8.05
Ehux + I 1	8.20	8.01
Ehux + I 2	8.17	8.01
Ehux + I 3	8.23	8.01

Table S3. Average nutrient drawdown for all conditions with *E. huxleyi* during the incubation period. Nutrients concentrations were measured (*) at the beginning and on day 4. From day 4 to the end of the experiment data refers to estimates assuming constant drawdown per cell and *E. huxleyi* buildup. Total nutrients concentrations used, corresponds to estimates considering constant drawdown per cell and *E. huxleyi* number of cells at the end of the experiment.

	**Day 0 to 4* (µM)**	**Day 4 to 13/14 (µM)**	**Total used (µM)**	***Nutrients available on day 13/14 (µM)***
Nitrate drawdown				
Present	9.07<br>(+/- 0.7)	45.06<br>(+/-17.58)	54.14	*26.65*
Future	17.79<br>(+/- 1.27)	13.33<br>(+/- 8.81)	31.12	*53.26*
Phosphate drawdown				
Present	0.84<br>(+/- 0.04)	4.19<br>(+/-1,48)	5.03	*-0.45*
Future	2.10<br>(+/- 0.13)	1.59<br>(+/- 1)	3.69	*1.12*

Table S4. Results of fermentation of carbohydrates by strains *I.abyssalis* and *Brachybacterium* sp.. Change in colour was expressed as (++) for clear positive, (+) for positive and (−) for negative reaction.

Carbon source (1%)	*I. abyssalis*	*Brachybacterium* sp.
Glucose	++	++
Fructose	++	++
Maltose	++	++
Trehalose	++	++
Ribose	++	-
Xylose	++	-
Dextrin	++	-
Starch	++	-
Lactose	+	-
Galactose	-	-
Arabinose	-	-
Rhamnose	-	-
Sucrose	-	-
Raffinose	-	-
Mannitol	-	-
Sorbitol	-	-
Inulin	-	-

Table S5. Percentage of particulate matter attributed to each species in the co-cultures based on average cellular quotas from the single cultures (white) or estimated for each condition (grey).

	Present						High CO_2					
	$TPC_{E.\ Huxleyi}$	$TPC_{bacteria}$	Total	$TPC_{E.\ Huxleyi}$	$TPC_{bacteria}$	Total	$TPC_{E.\ Huxleyi}$	$TPC_{bacteria}$	Total	$TPC_{E.\ Huxleyi}$	$TPC_{bacteria}$	Total
Brachybacterium sp.		80	80		100	100		191	191		100	100
		157	157		100	100		44	44		100	100
		90	90		100	100		68	68		100	100
I. abyssalis		131	131		100	100		96	96		100	100
		194	194		100	100		105	105		100	100
		58	58		100	100						
E. huxleyi	118		118	100		100	88		88	100		100
	83		83	100		100	56		56	100		100
	106		106	100		100	116		116	100		100
E + B	111	27	138	73	-11	62	108	27	135	73	-8	65
	65	4	69	96	35	131	78	11	90	89	22	110
	90	30	120	70	10	80	91	17	108	83	9	92
E + I	77	35	111	65	23	89	106	8	114	92	-6	86
	92	12	105	88	8	95	101	9	111	91	-1	89
	89	24	114	76	11	86	93	7	100	93	7	100
**Average Co-cultures**												
E + B	89	20	109	80	11	91	92	18	111	82	8	89
E + I	86	24	110	76	14	90	100	8	108	92	0	92
			10			*-10*			*9*			*-9*

Genome properties and phylogeny

In this study, the draft genome of two different strains, *Brachybacterium* sp. PhyBa_CO2_2 and *Idiomarina abyssalis* PhyBa_CO2_1, isolated from surface samples off the coast of Terceira and from an *E. huxleyi* strain isolated from surface water off Biscoitos, respectively, were analyse. The total size of the genome of *Brachybacterium* sp. PhyBa_CO2_2 was 3.6 Mbp (82 contigs) with an average GC content of 72.1%. For Idiomarina abyssalis PhyBa_CO2_1, the assembly generated 12 contigs comprising of 2.6 Mbp of total size with an average GC content of 47.1% (Table S9). In *Brachybacterium* sp. PhyBa_CO2_2 was predicted a total of 3312 genes, including 3257 protein-coding genes (CDSs), 5 rRNA genes and 50 tRNA genes. The number of total genes identified in *Idiomarina abyssalis* PhyBa_CO2_1 was 2534, including 2485 CDS genes, 3 rRNA genes and 47 tRNA genes (Table S9).

Based on 16S rRNA gene analysis, the strain *Idiomarina abyssalis* PhyBa_CO2_1 shared high sequence similarities with others *Idiomarina* sp. strains, including *Idiomarina abyssalis* strain MT141 and strain MCCC 1A02679 (Figure S1). The strain *Brachybacterium* sp. PhyBa_CO2_2 did not share high sequence similarity with others *Brachybacterium* sp. strains (Figure S2).

Genome-wide phylogeny of strains *Brachybacterium* sp. PhyBa_CO2_2 and *Idiomarina abyssalis* PhyBa_CO2_1 and theirs closest representative strains were compared by using ANI, ANI-Blast (ANIb) and ANI-MUMmer (ANIm). The ANI, ANIb and ANIm values between strain *Brachybacterium* sp. PhyBa_CO2_2 and most closely related specie *Brachybacterium* sp. AG952 were 96.18%, 95.48% and 96.03%, respectively. ANI, ANIb and ANIm values between *Idiomarina abyssalis* PhyBa_CO2_1 and members of the species *Idiomarina abyssalis* were in the range of 97.11–98.39%, 96.67–98.23% and 97.32–98.49%, respectively, surpassing the thresholds for species definition (Table S9).

Table S6. Genome properties and quality metrics of the strains sequenced in this study.

	**AU**	**ExT**
	Brachybacterium sp.	*Idiomarina abyssalis*
Size (Mbp)	3.6	2.6
Contigs	82	12
Scaffolds	76	9
%GC	72.1	47.1
N50 scaffolds (Mbp)	0.1	0.4
Completeness (%)	99.4	100
Contamination	0.3	0.2
Total gene count	3312	2534
CDS genes (%)	98.3	98.1
Number of rRNA genes (16S-23S-5S)	5	3
Number of tRNA genes	50	47
Genes in Pfams (%)	80.6	89.4
Genes in COG (%)	72.3	82.5

Table S7. Overview of phylogenetic comparisions between selected pairs of strains.

	Brachybacterium sp. PhyBa_CO2_2		
	ANI (mean) %	ANIb %	ANIm %
Brachybacterium sp. AG952	96,18	95,48	96,03
Brachybacterium sp. SW0106-09	96,05	95,51	96,00
Brachybacterium sp. sponge	95,81	94,96	95,69
Brachybacterium sp. HMSC06H03	95,80	95,41	96,14

	Idiomarina abyssalis PhyBa_CO2_1		
	ANI (mean) %	ANIb %	ANIm %
Idiomarina abyssalis UBA2690	98,39	98,23	98,49
Idiomarina abyssalis UBA3100	98,37	98,17	98,39
Idiomarina abyssalis UBA2697	98,17	98,24	98,37
Idiomarina abyssalis UBA4213	98,17	97,81	98,18
Idiomarina abyssalis UBA5050	98,07	97,91	98,27
Idiomarina abyssalis UBA3602	98,07	97,84	98,22
Idiomarina abyssalis KJE-1	98,00	97,73	98,06
Idiomarina abyssalis KJE-3	97,99	97,72	98,06
Idiomarina abyssalis KJE-2	97,99	97,73	98,06
Idiomarina abyssalis KMM 227	97,52	98,45	98,73
Idiomarina abyssalis UBA1461	97,28	96,79	97,3
Idiomarina abyssalis UBA5042	97,21	96,81	97,36
Idiomarina abyssalis UBA3366	97,14	96,79	97,32
Idiomarina abyssalis UBA3616	97,11	96,67	97,32

Table S8. Number of genes associated with general COG functional categories.

Class	Functional categories	*Brachybacterium* sp. Phy-Ba_CO2_2 Relative Abundance	*Idiomarina abyssalis Phy-Ba_CO2_1* Relative Abundance
E	Amino acid transport and metabolism	0,107	0,093
G	Carbohydrate transport and metabolism	0,114	0,025
D	Cell cycle control, cell division, chromosome partitioning	0,010	0,012
N	Cell motility	0,000	0,037
M	Cell wall/membrane/envelope biogenesis	0,055	0,064
B	Chromatin structure and dynamics	0,000	0,001
H	Coenzyme transport and metabolism	0,045	0,055
V	Defense mechanisms	0,019	0,013
C	Energy production and conversion	0,065	0,072
S	Function unknown	0,086	0,088
R	General function prediction only	0,151	0,123
P	Inorganic ion transport and metabolism	0,069	0,062
U	Intracellular trafficking, secretion, and vesicular transport	0,010	0,038
I	Lipid transport and metabolism	0,035	0,042
F	Nucleotide transport and metabolism	0,035	0,027
O	Posttranslational modification, protein turnover, chaperones	0,034	0,056
L	Replication, recombination and repair	0,057	0,052
A	RNA processing and modification	0,001	0,000
Q	Secondary metabolites biosynthesis, transport and catabolism	0,020	0,020
T	Signal transduction mechanisms	0,038	0,095
K	Transcription	0,083	0,060
J	Translation, ribosomal structure and biogenesis	0,072	0,076

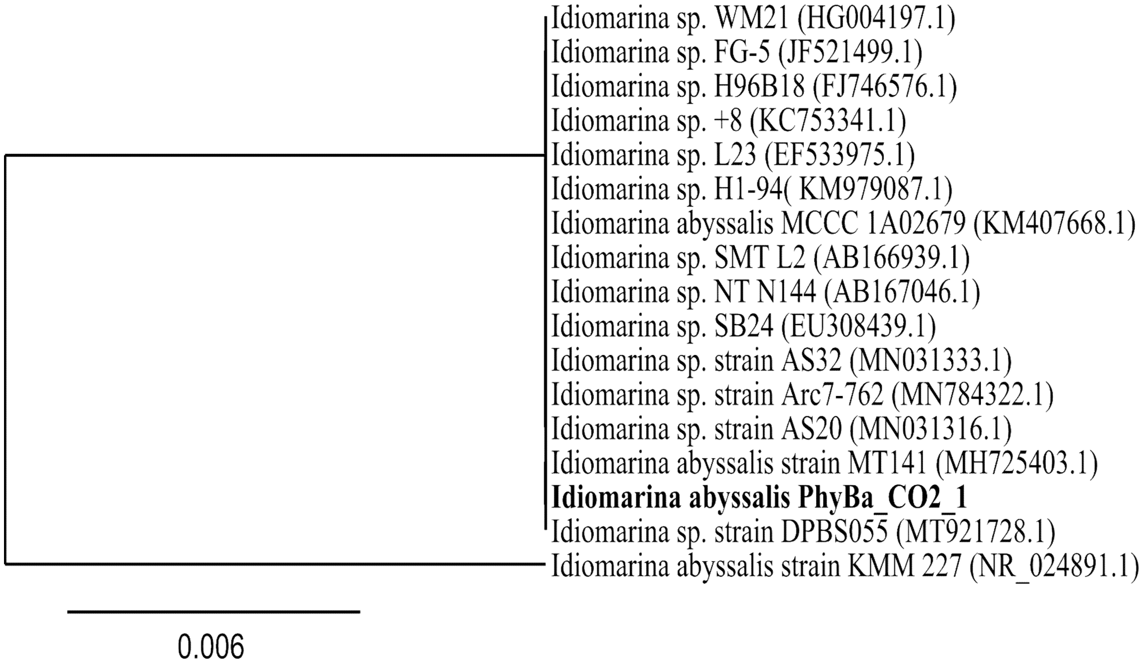

Figure S1. Phylogenetic tree inferred from 16S rDNA sequences, from *Idiomarina abyssalis* PhyBa_CO2_1 (in bold) and references strains (accession numbers in parenthesis).

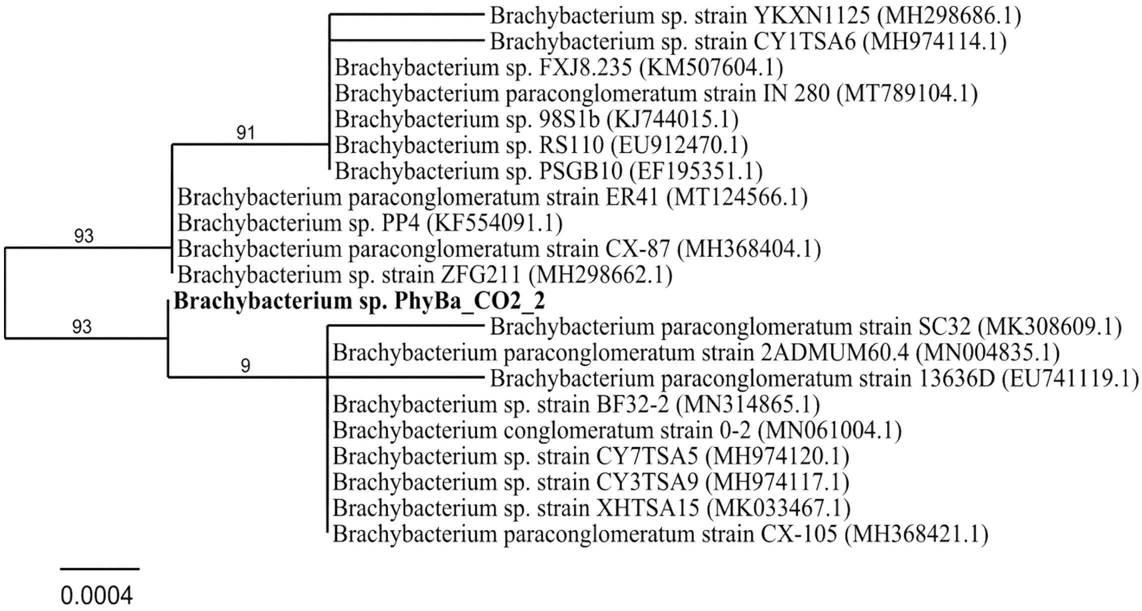

Figure S2. Phylogenetic tree inferred from 16S rDNA sequences, from *Brachybacterium* sp. PhyBa_CO2_2 (in bold) and references strains (accession numbers in parenthesis).
